# MIC13 of *Toxoplasma gondii*: Potential Gene for Vaccine Candidate—An In Silico Approach

**DOI:** 10.1155/japr/3114690

**Published:** 2025-09-26

**Authors:** Mahdi Khadem Mohammadi, Ali Dalir Ghaffari

**Affiliations:** ^1^Student Research Committee, Faculty of Medicine, Shahed University, Tehran, Iran; ^2^Department of Parasitology and Mycology, Faculty of Medicine, Shahed University, Tehran, Iran

**Keywords:** bioinformatics, MIC13, *Toxoplasma gondii*, vaccines

## Abstract

**Purpose:** Toxoplasmosis, which is the result of infection by *Toxoplasma gondii* (*T. gondii*), is a widespread parasitic disease that affects approximately one-third of the global population. Routine medications are not able to eradicate the parasites enclosed in cysts that reside inside the hosts that are infected. MIC13 is believed to have a significant function in facilitating the dissemination of the parasite throughout the host organism. The objective of this research was to utilize immunoinformatics techniques for antigenic analysis and structural prediction of the MIC13 protein, with the goal of identifying potential epitopes that could be used to create a vaccine for *T. gondii*.

**Materials and Methods:** The current research was aimed at describing the chemical and physical features, subcellular localization, potential epitopes for B- and T-cells, transmembrane domain, tertiary and secondary structures, and other attributes of the MIC13 protein.

**Results:** The results indicated that the MIC13 protein possesses a single N-glycosylation, 15 O-glycosylation regions, 70 phosphorylation sites, and five acetylation sites, with no transmembrane domains being detected within its structure. In terms of secondary structure, the MIC13 protein is composed of 27.99% alpha-helix, 16.45% extended strand, and 55.56% random coil elements. Additionally, various potential B- and T-cell epitopes were pinpointed for the MIC13 protein, suggesting its immunogenic properties. The assessment of antigenicity and allergenicity further confirmed that MIC13 is immunogenic but nonallergenic, making it a promising candidate for further study. Furthermore, the induction of IFN-*γ* and IL-4 highlighted the ability of related MHC-II molecules to interact with MIC13, indicating its potential role in immune responses. These findings shed light on the multifaceted nature of the MIC13 protein and its significance in immunological processes.

**Conclusion:** These findings suggest that MIC13 could serve as a key component in the creation of a successful vaccine targeting *T. gondii*. The results obtained from this research lay a solid foundation for future investigations and offer valuable insights for the creation of successful vaccines to combat both acute and chronic toxoplasmosis through diverse strategies.

## 1. Introduction

Toxoplasmosis, which results from infection with *Toxoplasma gondii* (*T. gondii*), is a widespread parasitic disease that affects approximately one-third of the global population [1]. The global prevalence of this parasite is believed to have affected over one billion individuals, making it a widespread and significant public health concern [2]. The parasite has three pathogenic forms: tachyzoites, which are known for their rapid growth; sporozoites located in oocysts; and bradyzoites found in tissue cysts. Humans can become infected by consuming oocysts excreted by cats, by eating infected meat containing tissue cysts, or by congenital transmission [3]. Tachyzoites are involved in the early stages of the disease, while bradyzoites are associated with the prolonged phase of the illness [4]. While *T. gondii* frequently results in asymptomatic infection, it has the potential to cause various neuropsychological symptoms, including hydrocephalus, vision impairment, cognitive disability, and brain inflammation. Additionally, severe complications can arise in infants with congenital infections and in individuals with compromised immune systems [5]. Additionally, *T. gondii* infection can cause considerable economic losses through stillbirths, abortions, and neonatal deaths in domestic animals, which serve as the primary source of transmission to humans [6, 7]. *T. gondii* infection stands as the third leading cause of illnesses from contaminated food necessitating inpatient care within the United States [8]. Furthermore, infection with *T. gondii* leads to financial setbacks due to miscarriages and the loss of newborn animals in agricultural settings, particularly among sheep and goat flocks [9–11]. Pyrimethamine and sulfadiazine (pyr-sulf) are currently the preferred treatment for toxoplasmosis, as they target the active stage of the infection. However, despite being the gold standard, notable failure rates still occur with this treatment [12]. Patients with HIV-AIDS who are prescribed cotrimoxazole frequently encounter numerous adverse effects, which raises concerns about its use. Furthermore, the medication is not recommended for pregnant women, as it can cross the placental barrier and pose risks to the fetus [13]. Drugs recommended for managing toxoplasmosis are effective in inhibiting the rapid multiplication of tachyzoites during the early stages of infection. However, these medications are not able to eradicate the parasites enclosed in cysts that reside inside the hosts that are infected [14]. Consequently, the development of suitable vaccines for this particular pathogen is a major objective due to its impact on the global health situation and the associated economic consequences within the animal husbandry industry [15]. Various methods have been employed thus far to prevent toxoplasmosis, including inactivated vaccines, vaccines that are live but weakened, and subunit vaccines [16]. By targeting specific epitopes, these vaccines can elicit a more targeted and robust immune response, potentially leading to improved protection against a variety of infectious agents [17]. In recent times, bioinformatics techniques have been employed to identify the possible epitopes for vaccine candidates recognized by T- and B-cells. Consequently, these approaches are appropriate for selecting dominant immunogenic epitopes [18]. The characterization of protein epitopes through bioinformatics methods can be beneficial for diagnostic purposes as well as the development of vaccines [19]. Various candidate antigens play a crucial role in inducing protective immunity against *T. gondii*. These antigens encompass secreted dense-granule proteins (GRAs), membrane-associated surface antigens (SAGs), micronemal proteins (MICs), and rhoptry proteins (ROPs) [20, 21]. MICs are involved in the complex mechanism of host cell invasion by the parasite. Among these proteins, MIC13 is believed to play a key role in facilitating the dissemination of the parasite throughout the host organism via interaction with the gut epithelium [22]. In contrast to most MICs, which are abundantly expressed during the tachyzoite stage to aid the parasite in infecting host cells, MIC13 shows comparatively low expression in tachyzoites but becomes markedly upregulated in bradyzoites or when the parasite experiences stress conditions [23]. Reflecting its expression profile, MIC13 appeared to have no involvement in tachyzoite invasion, replication, or egress and did not impact acute virulence in mice. Rather, it proved essential for ensuring optimal parasite growth during stress or conditions that promote bradyzoite formation [24]. The objective of this research was to utilize immunoinformatics techniques for antigenic analysis and structural prediction of the MIC13 protein, with the aim of identifying potential epitopes for the development of a *T. gondii* vaccine.

## 2. Materials and Methods

### 2.1. Sequence of Amino Acids

The National Center for Biotechnology Information (NCBI) databank in FASTA format was utilized in this research to conduct bioinformatics analysis of the MIC13 protein. The most comprehensive sequence of MIC13 (Gen Bank: EPT29482.1) was selected based on its length.

### 2.2. Chemical and Physical Features

The ExPASy ProtParam online tool was utilized to determine the theoretical chemical and physical properties of the *T. gondii* MIC13 protein. This included calculations of molecular weight (MW), isoelectric point (pI), and positively and negatively charged residues, as well as estimates for in vitro and in vivo half-life, aliphatic index, instability index (II), and grand average of hydropathicity (GRAVY) [25].

### 2.3. Identification of Transmembrane Domains and the Signal Peptide (SP)

The TargetP-2.0 online application aids in the recognition of thylakoid luminal transit peptide (luTP), SP, chloroplast transit peptide (cTP), and mitochondrial transit peptide (mTP) [26]. The transmembrane domains were predicted using the TMHMM-2.0 online server with the default settings. Additionally, the subcellular localization of MIC13 was forecasted through the PSORT II prediction tool [27].

### 2.4. Anticipation of Posttranslational Modification (PTM) Sites

The potential sites for PTMs of the proteins, such as phosphorylation, N-glycosylation, acetylation, and O-glycosylation, were predicted using the NetPhos 3.1 [28], NetNGlyc 1.0 [29], NetOGlyc 4.0 [30], and GPS-PAIL 2.0 [31] web servers, respectively. For NetNGlyc, the prediction was based on “all Asn residues,” while for acetylation, it was based on “all types.”

### 2.5. Anticipation of the Secondary Structural Features

The secondary structural elements of the investigated MIC13 protein were analyzed using the Garnier–Osguthorpe–Robson (GOR) IV server, which demonstrated a prediction accuracy of 64.4% [32]. Additionally, the prediction of disulfide bonds was conducted using the DI pro tool.

### 2.6. Creating a Three-Dimensional Model

Utilizing the SWISS-MODEL web-based database, a technique known as homology modeling was employed to produce the 3D representations of the MIC13 protein. This database is well known for its ability to forecast the three-dimensional structures of proteins [27, 33].

### 2.7. Enhancement and Authentication of the 3D Designed Configuration

To improve the accuracy of predicting protein systems based on templates, the best performing model obtained from SWISS-MODEL was selected and subsequently refined through GalaxyRefine. GalaxyRefine first reconstructs the side chains, repacks them, and then refines the entire structure through molecular dynamics simulations [34]. The protein's 3D conformation was confirmed through analysis with the Ramachandran plot utilizing the SWISS-MODEL software [35]. The Ramachandran plot provides a visual depiction of the energetically favorable regions of backbone dihedral angles corresponding to amino acid residues within a protein structure. This tool aids in understanding the conformational quality and stability of the protein model under analysis. Additionally, the model's general quality assessment can be conducted using ProSAweb, a web-based tool that evaluates the energy profile and potential structural errors [36].

### 2.8. Discontinuous (Conformational) and Continuous (Linear) B-Cell Epitopes of the MIC13 Protein

The B-cell epitopes for the MIC13 protein were forecasted through the utilization of multiple servers. Initially, the BCPREDS (B-cell epitope prediction server) predicted the continuous epitopes of B-cell by employing a combination of a support vector machine (SVM) approach and a subsequence kernel, yielding an output accuracy of 74.57% (the predicted B-cell epitopes were characterized with a set threshold of 0.80%) [37]. Subsequently, the Bcepred (B-cell epitope prediction) tool was employed to forecast the linear epitopes of B-cells based on physicochemical attributes. This online server can achieve a prediction accuracy of up to 58.70% at a threshold of 2.38, enabling users to predict B-cell epitopes by evaluating various chemical and physical properties, including accessibility, flexibility/mobility, hydrophilicity, exposed surface, polarity, and turns [16, 38]. Additionally, the ABCpred server, which stands for artificial neural network–based B-cell epitope prediction, was also employed in the analysis [16, 39]. The ABCpred server was designed with the aim of forecasting epitopes of B-cell within an antigen sequence by employing an artificial neural network (ANN). The predicted B-cell epitopes were characterized by a chain consisting of 16 amino acids, with a set threshold of 0.80%. Additionally, Bepipred linear epitope prediction [40], hydrophilicity [41], antigenicity [42], betaturn [43], surface accessibility [44], and flexibility [45] were predicted through the use of the immune epitope database (IEDB). Finally, the assessment of water solubility, antigenicity, and allergenicity was conducted using PepCalc, VaxiJen v2.0, and AllergenFP v1.0 [46] internet-based servers, respectively. Subsequently, ElliPro was employed to predict noncontinuous epitopes of B-cell [47].

### 2.9. Epitope Forecasting for Major Histocompatibility Complex (MHC)-I and -II Molecules

The IEDB online service was utilized to forecast the binding affinity of MIC13 peptides to the major MHC-II and MHC-I molecules based on the BALB/c mouse strain alleles. The mouse MHC-I molecules H2-Kd, H2-Ld, H2-Kb, H2-Db, H2-Dd, and H2-Kk were specifically chosen for this analysis, while the MHC-II molecules H2-IAb, H2-IEd, and H2-IAd were also involved in the prediction process. Peptide binding prediction was conducted using 10-mer peptides for MHC-I molecules and 15-mer peptides for MHC-II molecules, following the recommended method by IEDB. The peptides were sorted by percentile rank to determine their binding potential to the respective MHC molecules accurately. Subsequently, the antigenicity and the capability to induce IFN-*γ* and IL-4 for each epitope were forecasted using IFNepitope, VaxiJen v2.0, and IL-4-pred web servers.

### 2.10. Forecasting Epitopes for Cytotoxic T Lymphocytes (CTLs)

To identify peptides capable of stimulating a cytotoxic T-cell response, the CTLpred server was utilized [48]. The forecasting process relied on a combined technique [49]. A combined approach was applied using a cutoff value to distinguish between epitopes and nonepitopes.

### 2.11. Assessment of Antigenic Properties, Allergic Potential, and Solubility

VaxiJen v2.0 [50] and ANTIGENpro [51] servers were utilized in order to forecast the antigenicity of the MIC13 protein. ANTIGENpro serves as a tool for predicting protein antigenicity that is sequence-based, alignment-free, and independent of the pathogen being studied. The VaxiJen v2.0 server introduces a novel method for antigen forecasting by utilizing autocross covariance (ACC) to transform sequences of proteins into standardized vectors of key amino acid characteristics [52]. The prediction of allergenicity by the server was achieved through the utilization of six distinct methods. A hybrid approach, combining ARPs BLAST, SVMc, MAST, and IgE epitope, demonstrated an accuracy of 85% at a threshold of −0.4. Subsequently, the SOLpro server was employed to forecast the solubility of protein upon overexpression [53]. SOLpro utilizes a two-step SVM architecture and various primary sequence representations to forecast the solubility of a protein in *Escherichia coli* (*E. coli*) during overexpression.

### 2.12. Simulation of the Immune System

The CImmSim online server was utilized to assess the immune responses of the MIC13 [54]. The server simulates the antibody-mediated (B-cell) and cellular (T-cell) immunological responses of the host immune system toward the vaccine structure. Immune response prediction is based on the stimulation of three vital anatomical systems in mammals: the bone marrow, thymus, and lymph node. In accordance with prior research, the vaccine construct involved the administration of three doses at 4-week intervals [54]. The default values for the simulation parameters were established at distinct time intervals of 1, 84, and 168 time steps, correspondingly. Key parameters included a simulation volume of 50 and 1050 simulation steps. Furthermore, an LPS-free vaccine injection was administered with a random seed value of 12,345.

## 3. Results

### 3.1. Characteristics Pertaining to Both Physical and Chemical Aspects

The MIC13 protein's amino acid sequence was retrieved from the NCBI database in FASTA format with the accession number EPT29482.1. The protein was found to consist of 468 amino acid residues, with a MW of 51.45970 kDa and a theoretical pI of 6.05. Additionally, the MIC13 protein was observed to contain 63 negatively charged residues (Asp + Glu) and 57 positively charged residues (Arg + Lys). The total number of atoms in the protein was determined to be 7044, and its extinction coefficient at 280 nm in water was calculated to be 42005 M^−1^ cm^−1^ in water. Furthermore, the half-life of the MIC13 protein was estimated to be 30 h for reticulocytes of mammals in vitro, greater than 20 h for yeast in vivo, and greater than 10 h for *E. coli* in vivo. The protein's II was calculated to be 44.79, indicating that the protein falls into the category of unstable proteins. Moreover, the aliphatic index and GRAVY of the MIC13 protein were found to be −0.565 and 61.79, respectively. The negative GRAVY score suggests that the MIC13 protein has a hydrophilic nature.

### 3.2. Transmembrane Domain and SP

**Figure 1:**
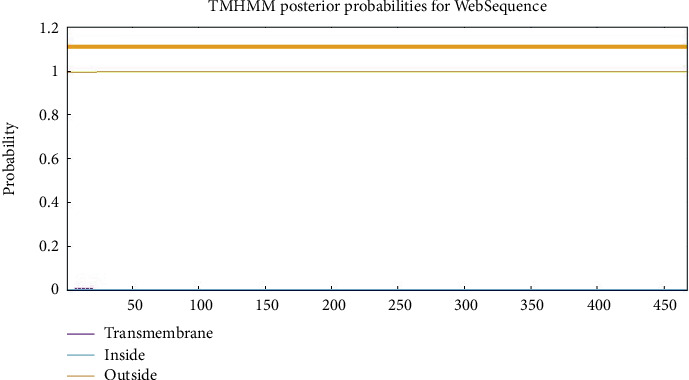
Analysis of the transmembrane helical domains in MIC13.

The findings from the TargetP-2.0 server indicated the presence of a SP within the protein's sequence. Furthermore, the results obtained from the TMHMM server v.2.0 revealed that the MIC13 sequence did not contain any transmembrane domain, as illustrated in Figure 1. Additionally, PSORT II was used to predict the subcellular localization of MIC13, with 44.4% being extracellular, including the cell wall, 33.3% cytoplasmic, 11.1% mitochondrial, and 11.1% nuclear.

### 3.3. Projecting the Positions of PTM Sites

**Figure 2:**
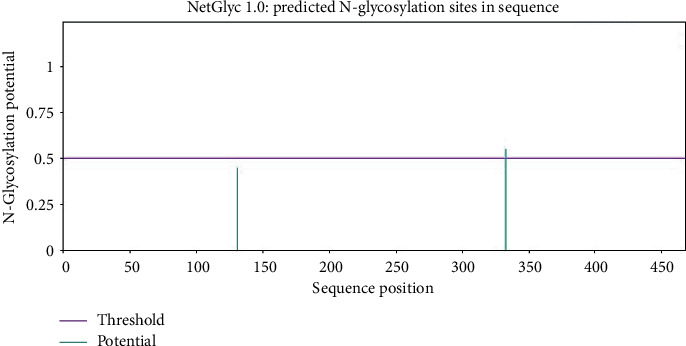
The results obtained from the NetNGlyc server regarding the N-glycosylation sites of MIC13. N-linked glycosylation potential across the polypeptide chain, determined using the NetNGlyc 1.0 server. The plot highlights multiple residues with scores exceeding the 0.5 predictive threshold (dashed line), indicating potential sites for N-glycosylation.

**Figure 3:**
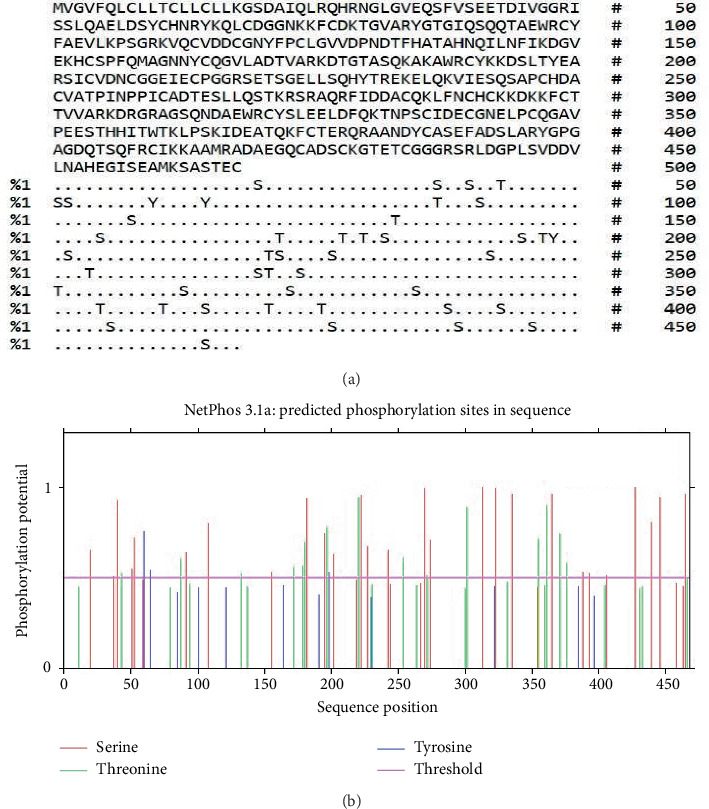
The output regarding phosphorylation sites on MIC13 that was obtained through the NetPhos server. Comprehensive mapping of potential phosphorylation sites on serine, threonine, and tyrosine residues, as predicted by the NetPhos 3.1 algorithm. The multiplicity of sites surpassing the 0.5 phosphorylation potential threshold suggests that MIC13 is a substrate for extensive regulatory phosphorylation.

**Figure 4:**
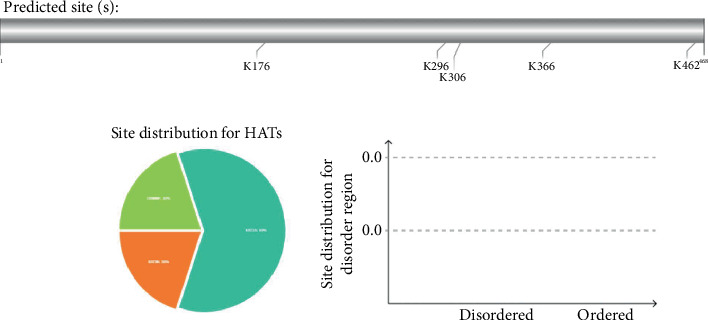
Anticipated acetylation sites determined via GPS-PAIL 2.0 server employing a medium threshold.

The MIC13 sequence contained a single N-glycosylation site, as illustrated in Figure 2. A total of 15 O-glycosylation regions were identified within the MIC13 protein structure. Furthermore, MIC13 was found to harbor 45 phosphorylation sites, with 27 being serine, 15 threonine, and three tyrosine residues, as depicted in Figure 3. Additionally, GPS-PAIL 2.0 analysis revealed the presence of five acetylation sites when utilizing a medium threshold, as shown in Figure 4 and Table 1.

### 3.4. Anticipation of Secondary and Tertiary Configurations

**Figure 5:**
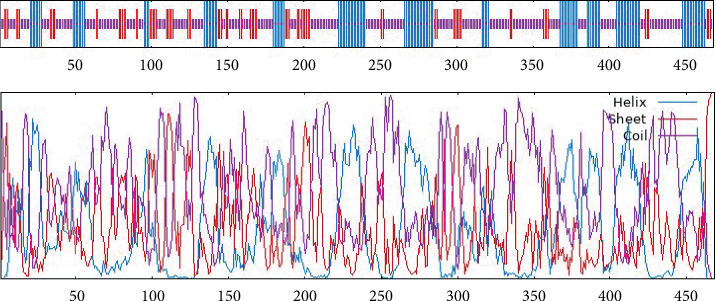
Visual representation of the predicted secondary structure of MIC13 utilizing the GOR IV web-based tool. The GOR IV prediction provides a complementary assessment. Both models converge on a structural landscape characterized by a high proportion of random coils and intrinsically disordered regions, interspersed with shorter, defined *α*-helical and *β*-sheet domains.

The MIC13 protein's secondary structure was analyzed utilizing the GOR4 online service, which is capable of predicting structural components like extended chain, random coil, and alpha-helix. The results obtained from the GOR4 server revealed that the MIC13 sequence consisted of 55.56% random coil (260/468), 27.99% alpha-helix (131/468), and 16.45% extended strand (77/468) (Figure 5). Additionally, the predicted number of bonds was 15. Cysteines at the following positions are predicted to form the disulfide bond: 8, 12, 15, 61, 69, 77, 99, 114, 118, 124, 154, 165, 190, 204, 208, 214, 247, 261, 284, 290, 292, 299, 336, 346, 375, 386, 424, 428, 434, and 468. The findings from the DI pro server were also presented (Table 2).

### 3.5. Prognostication and Evaluation of Three-Dimensional Model

**Figure 6:**
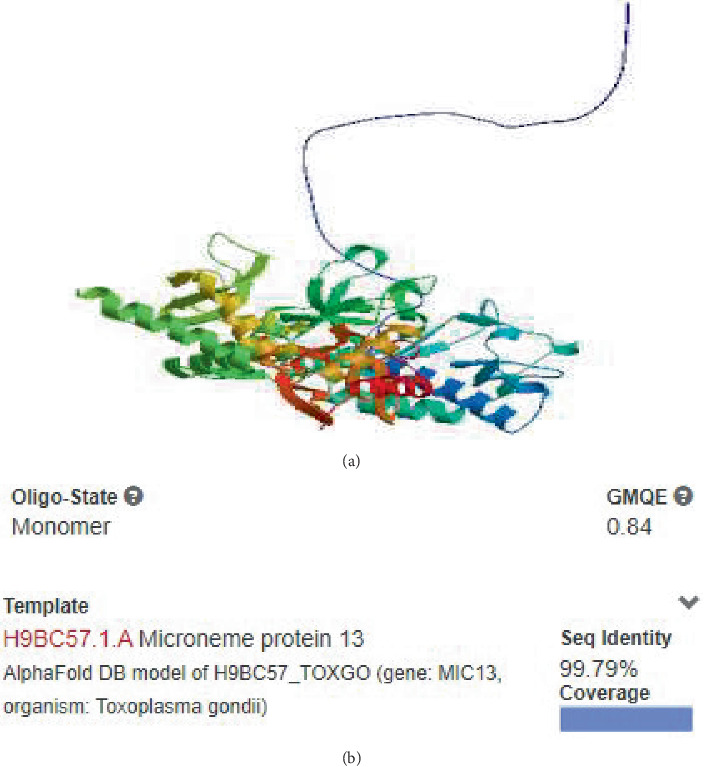
Output of SWISS-MODEL server. (a) Created 3D model utilizing the SWISS-MODEL server. (b) Identity, similarity, and coverage of sequence.

The SWISS-MODEL platform was employed to generate three-dimensional models of the MIC13 protein. After model prediction, four distinct 3D models were created, and the model most similar to the MIC13 sequence was selected. This model displayed a sequence identity of 99.79% compared to the other generated models. The outcomes obtained from SWISS-MODEL are presented in Figure 6, showcasing various aspects such as sequence coverage and identity, local quality estimate, model template alignment, global quality estimate, and the 3D model constructed for the MIC13 protein.

### 3.6. Enhancement and Authentication of the 3D Configuration

**Figure 7:**
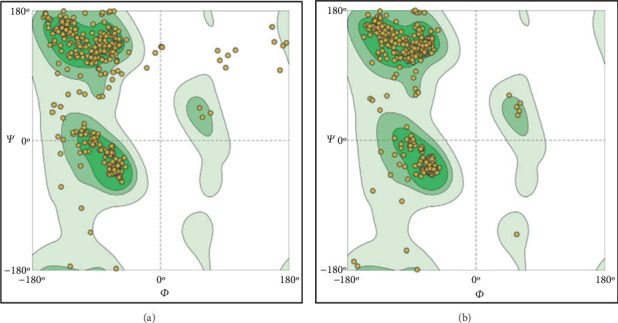
The analysis of the Ramachandran plot utilizing the SWISS-MODEL server in (a) the initial model and (b) the model after refinement.

**Figure 8:**
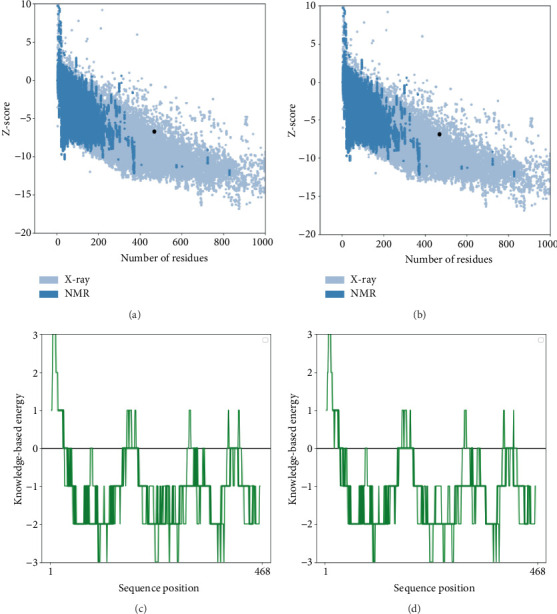
Validation of MIC13 protein's 3D structure was conducted through analysis of Ramachandran plot. (a) The *Z*-score plot for the initial 3D structure of the predicted vaccine yielded a value of −6.69. (b) The *Z*-score plot for the 3D structure after refinement yielded a value of −6.83. (c) The graph of knowledge-based energy according to sequence position, which is related to 3D structure of the predicted vaccine before refinement. (d) The graph of knowledge-based energy according to sequence position, which is related to 3D structure of the predicted vaccine after refinement.

The tertiary structures were refined using the GalaxyRefine program, resulting in an improvement in the quality of the 3D structures as evaluated by the Ramachandran plot (Figure 7) and ProSAweb analysis. The initial ProSAweb results showed a *Z*-score of −6.69, with a significant number of residues falling within the favored region (Figure 8a). After refinement, the overall model quality was enhanced, with a *Z*-score of −6.83 (Figure 8b). Additionally, the graphs illustrating knowledge-based energy based on sequence position before and after refinement are presented (Figure 8c,d).

### 3.7. Discontinuous and Continuous B-Cell Epitopes of the MIC13 Protein

**Figure 9:**
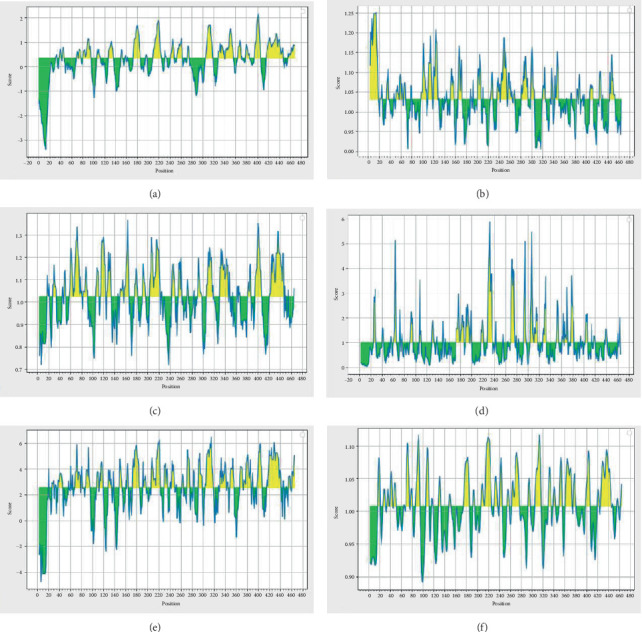
Propensity scale graphs for MIC13 protein. (a) Bepipred linear epitope prediction. (b) Antigenicity. (c) Betaturn. (d) Surface accessibility. (e) Hydrophilicity. (f) Flexibility. The *Y*-axes of the graphs display the average score of each residue within a defined window, with the *X*-axes indicating the positions of the residues in the sequence. The tables present the calculated scores for each residue. Residues with higher scores may indicate a higher probability of being part of an epitope, as shown by their yellow color on the graphs. Conversely, regions colored green below the threshold indicate unfavorable areas in terms of the properties of interest.

**Figure 10:**
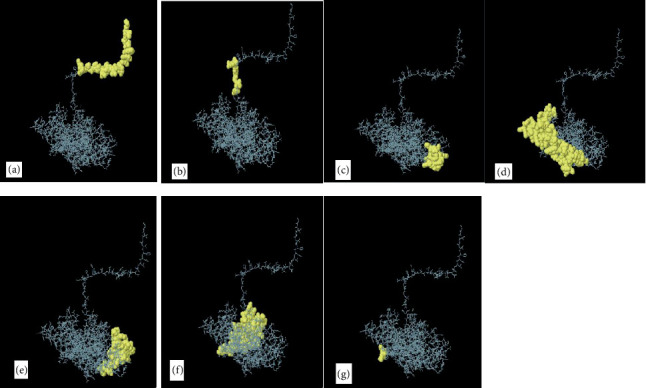
(a–g) 3D arrangement of discontinuous B-cell epitopes within the sequence of MIC13. The majority of the polyprotein is illustrated with gray sticks, while the discontinuous areas of the B-cell epitopes are illustrated with yellow surfaces.

BCPREDS was specifically employed for the prediction of B-cell epitopes, resulting in nine predicted epitopes with high scores presented in Table 3. The higher threshold score indicates stronger binding affinity and specificity for the predicted epitopes. This data is essential for comprehending the possible interactions between the epitopes and their corresponding antibodies. Bcepred was utilized to categorize the continuous B-cell epitopes based on various features such as exposed layer, accessibility, turns, mobility, antigenic propensity, polarity, and hydrophilicity. The outcomes of the Bcepred server are presented in Table 4 for further analysis. These epitopes perform a crucial function in determining the antigenic properties of the MIC13 protein, shedding light on the potential immune responses triggered by these regions. The comprehensive analysis provided by these servers aids in the exploration of novel vaccine candidates and therapeutic targets.  Table 5 summarizes the results from the ABCpred server, indicating B-cell epitopes of 16 residues in length, with all listed peptides exceeding the specified threshold value. The predicted B-cell epitopes are classified based on scores produced by the trained recurrent neural network, where higher scores correspond to a greater likelihood of being true epitopes. We utilized the IEDB online server to determine the average scores of betaturn, antigenicity, hydrophilicity, Bepipred linear epitope prediction, surface accessibility, and flexibility for the MIC13 protein, which were found to be 2.529, 1.031, 1.024, 0.269, 1.008, and 1.000, respectively, as illustrated in Figure 9. Furthermore, the ElliPro web server predicted seven discontinuous epitopes related to B-cells, with scores ranging from 0.593 to 0.966, as shown in Figure 10 and Table 6.

### 3.8. Forecasting Epitopes for MHC Types I and II

The IEDB online tool was employed to predict MHC-I and MHC-II binding epitopes of the MIC13 protein across various mouse alleles. Through bioinformatics analysis, T-cell epitopes of MIC13 were identified to exhibit strong binding capabilities with MHC-I and MHC-II molecules. Epitopes for the MIC13 protein were chosen according to their lowest percentile ranks, suggesting a stronger attraction to the receptor molecule. The minimum percentile ranks for each MHC allele associated with MIC13 are reported in Tables 7 and 8 for reference.

### 3.9. Anticipating CTL Epitopes

Forecasting CTL epitopes was conducted through the utilization of the CTLpred server, with the subsequent selection of appropriate epitopes being carried out by CTLpred. Additional information is available in Table 9.

### 3.10. Analysis of Antigenic Properties, Allergic Potential, and Solubility

The MIC13 protein exhibited a high predicted antigenicity, with values of 0.9663 by ANTIGENpro and 0.5624 by VaxiJen v2.0 (threshold = 0.5). These results from both prediction tools indicate the immunogenic potential of the MIC13 protein. Furthermore, the AlgPred server analysis confirmed that the MIC13 protein is nonallergenic, providing additional insights into its safety profile for potential applications. Additionally, the solubility of the MIC13 protein following overexpression in *E. coli* was determined to be 0.5925 by the SOLpro server, indicating its potential for downstream purification and characterization.

### 3.11. Immune System Modeling

**Figure 11:**
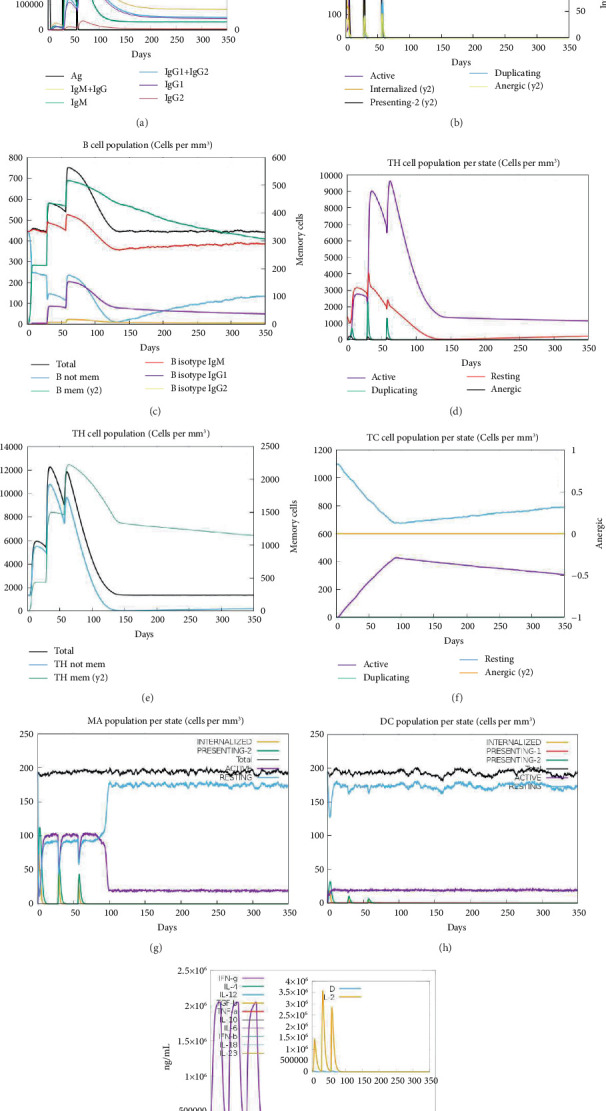
The immune simulation server was utilized to model and analyze the immune responses triggered by the epitope MIC13. (a) Antigen and immunoglobulin, (b) B-cell population per state, (c) B-cell population, (d) CD_4_^+^ T-cell population per state, (e) CD_4_^+^ T-cell population, (f) CD_8_^+^ T-cell population per state, (g) macrophage population per state, (h) dendritic cell population per state, and (i) cytokine and interleukin production.

The antibody response exhibited a substantial increase after each administration and following encounters with the epitope-derived peptide vaccine. The predominant immune reaction was identified by heightened levels of IgM antibodies. Additionally, heightened expressions of antibodies, such as IgG + IgM, IgM, and IgG1 + IgG2, were linked to an augmented B-cell density, followed by a reduction in the concentration of antigens and a noteworthy rise in memory B-cells (Figures 11a, 11b, and 11c). The study also noted the development of tertiary and secondary immune reactions, accompanied by memory, which led to a following rise in cytotoxic T-cell and helper T-cell density (Figures 11d, 11e, and 11f). Throughout the course of the doses, it was observed that levels of IL-2 and IFN-*γ* rose after vaccination (Figures 11g, 11h, and 11i). As memory continued to increase, the population of T-cells became significantly more accessible, while the remaining immune cells remained relatively constant. These findings provide evidence that the vaccine design has the capacity to induce substantial anti-LD responses of the immune system.

## 4. Discussion

*T. gondii* infections present a significant concern for public health and have a significant global economic impact [55, 56]. The creation of a reliable and secure vaccine is essential for safeguarding against toxoplasmosis [57]. The initial step in developing a potentially effective vaccine involves identifying the antigens of the parasite that may provide immunoprotection. This crucial phase sets the foundation for further vaccine development. By pinpointing the key antigens, researchers can focus their efforts on creating a vaccine that triggers a robust immune response against the parasite [58]. Bioinformatics tools have emerged as a critical component in the creation of potent vaccines, being widely employed in the evaluation of protein epitopes, functions, and structures [6]. This study examined various characteristics of the MIC13 protein through the application of multiple bioinformatics resources to create a successful vaccine for *T. gondii*. Analysis of the MIC13 amino acid sequence indicated the presence of 468 residues with a MW of 51.45970 kDa. This suggests that the protein possesses favorable antigenic properties, as antigens with a MW below 5–10 kDa are generally considered to be less effective as immunogens [52]. The stability of the protein was assessed through the calculation of the II, revealing that the MIC13 protein had an II of 44.79, indicating its classification as an unstable protein due to exceeding the threshold of 40 for protein instability. Furthermore, the GRAVY value and aliphatic index for the MIC13 sequence were found to be 61.79 and −0.565, respectively. A high aliphatic index suggests that the protein is more stable across a variety of temperature levels, indicating that MIC13 is thermostable. Additionally, the negative GRAVY value signifies that the protein is hydrophilic, enabling it to effectively interact with water molecules in its surroundings. It is widely recognized that PTMs have a crucial role in controlling cellular functions [57]. The proteins' PTM sites, which include phosphorylation, O-glycosylation, acetylation, and N-glycosylation, were anticipated through the use of different web servers. The investigation revealed that the protein lacks transmembrane domains, making it accessible to antigen-presenting cells for the activation of B-cell, CD_4_^+^ T-cell, and CD_8_^+^ T-cell reactions. It is crucial to emphasize the significance of analyzing the protein's secondary structure in the forecasting of its tertiary structure, which involves the incorporation of specific restraints like beta-turn or alpha-helix. In this study, the GOR4 software was utilized as it is a widely used and reliable tool for evaluating the vaccine's secondary structure. Notably, the beta-turn and alpha-helix are situated in the internal regions of the protein, which have high hydrogen bond energy, contributing to the stability of the protein's structure and enhancing interactions with antibodies [59]. Furthermore, the DI pro server was employed to predict four disulfide bonds within the MIC13 sequence. These bonds, also called disulfide bridges, are crucial for the protein's structural integrity and functional characteristics [60]. Disulfide bonds with fewer than five sequences are more predictable compared to those with more than five bonds. Additionally, precise forecasting of disulfide bonds may decrease the range of possible molecular shapes, thereby enhancing the modeling of protein 3D structures and protein folding [61]. It is crucial to have a comprehensive understanding of protein structures in order to discern the intricate relationships between structure and function [27, 58, 62]. The tertiary conformation of the MIC13 protein was predicted in this investigation through the utilization of the SWISS-MODEL tool. An additional important factor in the prediction of protein structure involves structural validation, with the Ramachandran plot serving as a tool to evaluate the accuracy of experimental structures and to make predictions about the biological role of a protein [63]. The Ramachandran plot was generated utilizing the SWISS-MODEL method to verify the precision of the 3D model produced. Structural biology emphasizes the importance of detecting errors in both theoretical and experimental protein structure models, making model validation a critical step in the process. The assessment of the Ramachandran plot postrefinement indicated a top-notch 3D model, with the MIC13 protein demonstrating promising characteristics such as a significant proportion of residues in favored regions and a minimal percentage in the outer region. This suggests a reliable structural representation of the protein.

In the context of *T. gondii* infection, a robust immune response, involving both humoral and cell-mediated components, is triggered. This response is essential for combating the infection, underscoring the importance of understanding host–pathogen immune interactions [64]. The cell-mediated immune reaction plays a crucial role in combating both chronic and acute *T. gondii* infections. T-cells, such as CD_4_^+^ and CD_8_^+^ T-cells, along with cytokines like tumor necrosis factor-*α*, IL-2, IFN-*γ*, and IL-12, are responsible for providing defensive immunity. Additionally, cytokines like IL-5, IL-4, and IL-10 are essential in controlling immune reactions [65, 66]. However, the generation of B-cell antibodies is equally vital for sustaining long-lasting protection against this particular disease [67]. The epitope, a component of an antigen, is recognized by CD_4_^+^ and CD_8_^+^ T-cells, B-cells, and various molecules within the host's immune system. The examination of epitopes plays a vital function in determining the antigenicity and specificity of protein antigens, aiding in understanding the structure and function of the target antigen [27, 58]. Our study was aimed at identifying immunodominant epitopes within MIC13 protein sequences that have the ability to trigger both cellular and humoral immune responses. To enhance the dependability and precision of our predictions, we utilized various methods and online servers to predict B-cell, CD_4_^+^ and CD_8_^+^ T-cell epitopes. The results from the analysis of linear epitopes of B-cells showed that the MIC13 protein contained favorable epitopes with appropriate indices, as determined by BCPREDS, ABCpred, IEDB, and Bcepred web-based servers. For instance, we used a Bcepred server to forecast linear epitopes of B-cells within protein sequences. The predictive accuracy of Bcepred for models utilizing various features ranged from 52.92% to 57.53%, indicating its reliability in identifying B-cell epitopes [38]. The ABCpred database utilizes an ANN to forecast epitopes of B-cells within an antigen sequence. Through the implementation of a recurrent neural network, the server is capable of predicting epitopes with a precision rate of 65.93% [39]. An essential step in conducting in silico studies involves identifying conformational epitopes essential for the interaction between antigens and antibodies. The ElliPro web server anticipated seven discontinuous B-cell epitopes. The IEDB online server was used to assess the inhibitory concentration (IC_50_) values of peptides binding to MHC-I and MHC-II molecules for MIC13. The epitopes of T-cells on MIC13 demonstrated strong attachment to MHC-I and II molecules based on the IEDB results. Notably, lower percentile ranks (or IC_50_ values) show an increased level of affinity, suggesting a superior T-cell epitope, or the opposite. It is crucial to determine the specific type of T-cell-based immune reaction that is involved in order to develop a successful toxoplasmosis vaccine. Therefore, in the creation of an appropriate vaccine for toxoplasmosis, it is of utmost importance to identify the particular T-cell-based immune reaction that helps greatly in fighting off the infection [62, 64]. So, we utilized the CTLpred server to forecast CD_8_^+^ T-cell epitopes and selected the Top 10 epitopes for the MIC13 protein. The CTLpred server employs a specialized technique for predicting CTL epitopes, which performs a crucial function in the development of the vaccine.

The identification of allergenic proteins holds significant importance in the present day, primarily because of the utilization of modified proteins in the manufacturing of biopharmaceuticals, genetically modified foods, therapeutic techniques, and other related applications [52]. Analysis of antigenicity using VaxiJen and ANTIGENpro, along with allergenicity via AlgPred, indicated that the MIC13 protein is immunogenic and nonallergenic. The results of the immune simulations closely mirrored the actual immune reactions to peptide antigens. With repeated exposure to the MIC13, an overall increase was observed in the generated immune reactions. The findings unequivocally demonstrate that memory B-cells persisted for a few months, and CD_4_^+^ T-cells were notably activated, indicating their maturation. A fascinating discovery was the continual elevation in levels of IL-2 and IFN-*γ* after the initial dosage, even with several administrations of the antigen. This analysis implies a large population of helper T-cells, which in turn bolsters a robust antibody-mediated immune reaction [68, 69].

Similar to our study, numerous studies focusing on MIC-based vaccines have utilized bioinformatics servers and diverse tools to forecast potential T-cell and B-cell epitopes, aiming to create a highly effective vaccine against toxoplasmosis. Although our investigation was restricted to in silico analysis, evidence from previous studies on this gene suggests that MIC proteins can enhance survival in mice. In studies where mice were immunized with a DNA vaccine encoding *T. gondii* MIC3 and ROP18, the vaccinated animals exhibited prolonged survival against toxoplasmosis and developed more robust humoral and Th1-type cellular immune responses [70]. Also, similar to Onile et al.'s study [71], which was conducted in MIC proteins (including MIC6, MIC1, MIC4, MIC8, MIC2, MIC15, MIC3, MIC16, and MIC13), our findings revealed that MIC13 displayed high immunogenicity scores, and in silico analyses identified several potential epitopes within MIC proteins, highlighting the usefulness of these approaches for predicting immunogenic epitopes.

Bioinformatics serves as a powerful tool for analyzing protein data, identifying immunogenic epitopes, and ultimately generating recombinant proteins [72]. While such approaches are essential for accelerating and improving the selection of potential vaccine candidates, in silico methods have inherent limitations in predicting the physicochemical characteristics, structural features, and immunogenicity of chimeric peptides [72]. Therefore, the effectiveness of any recombinant vaccine design must still be validated through experimental assays and animal studies. By utilizing both computational and experimental methods, researchers can gain a comprehensive understanding of the protein's ability to be considered as a possible vaccine option.

## 5. Conclusion

The current research was aimed at describing the chemical and physical features, subcellular localization, potential epitopes for B- and T-cells, transmembrane domain, secondary and tertiary structure, and other attributes of the MIC13 protein. MIC13 exhibited high flexibility, hydrophilicity, surface accessibility, and antigenicity scores, indicating its capability as a potential vaccine candidate against *T. gondii*. Furthermore, the bioinformatics analysis conducted to forecast epitopes of CD_8_^+^ T-cells, CD_4_^+^ T-cells, and B-cells revealed several promising epitopes within the MIC13 protein sequence. These findings suggest that MIC13 could serve as a key component in the creation of a successful vaccine targeting *T. gondii*. The utilization of in silico tools for structural and functional predictions of MIC13 proved to be beneficial in reducing risks related to experimental studies. The results obtained from this research lay a solid foundation for future investigations and offer valuable insights for developing effective vaccines against both acute and chronic toxoplasmosis.

## Figures and Tables

**Table 1:** Identification of acetylation sites conducted utilizing GPS-PAIL 2.0 server with medium threshold.

**ID**	**Position**	**Peptide**	**Score**	**Cutoff**
1	176	LADTVARKDTGTASQ	1.464	1.382
2	296	NCHCKKDKKFCTTVV	1.431	1.343
3	306	CTTVVARKDRGRAGS	1.493	1.343
4	366	TWTKLPSKIDEATQK	1.536	1.348
5	462	EGISEAMKSASTEC	2.645	0.42

**Table 2:** Forecasted disulfide bonds utilizing DI pro.

**Bond index**	**Cys1_position**	**Cys2_position**
1	69	77
2	424	434
3	247	261
4	336	346
5	292	299
6	428	468
7	208	214
8	99	114
9	190	204
10	118	124
11	375	386
12	154	165
13	15	61
14	284	290
15	8	12

**Table 3:** Predictions of linear B-cell epitopes from the MIC13 protein generated using BCPRED.

**No.**	**Position**	**Epitope**	**Score**	**VaxiJen score**	**Allergenicity**	**Water solubility**
1	395	ARYGPGAGDQTSQFRCIKKA	1	0.2832	Allergen	Good
2	204	CVDNCGGEIECPGGRSETSG	0.992	0.9981	Allergen	Good
3	247	CHDACVATPINPPICADTES	0.962	1.0950	Allergen	Poor
4	343	ELPCQGAVPEESTHHITWTK	0.928	0.4513	Allergen	Good
5	304	ARKDRGRAGSQNDAEWRCYS	0.91	1.6684	Nonallergen	Good
6	176	KDTGTASQKAKAWRCYKKDS	0.865	1.0082	Allergen	Good
7	81	GVARYGTGIQSQQTAEWRCY	0.848	0.4484	Allergen	Poor
8	371	TQKFCTERQRAANDYCASEF	0.842	−0.0007	Allergen	Good
9	226	LSQHYTREKELQKVIESQSA	0.801	0.5202	Allergen	Good

**Table 4:** Forecasted B-cell epitopes utilizing physicochemical properties from the Bcepred server.

**Prediction parameter**	**Epitope sequence**
Accessibility	CDGGNKK, QSQQTAE, CVDDCGN, KDGVEKH, DTVARKDTGTASQKAK, RCYKKDS, VDNCGGE, ECPGGRSETSGE, ESQSAPC, QSTKRSRAQR, NCHCKKDKK, ARKDRGRAGSQNDAE, QKTNPSCIDECGNE, PEESTHH, TERQRAANDYCASE, GPGAGDQTSQ, RADAEGQCADSCKGTETCGGGRSR, and KSASTEC
Hydrophilicity	CDGGNKK, QSQQTAE, CVDDCGN, KDGVEKH, DTVARKDTGTASQKAK, RCYKKDS, VDNCGGE, ECPGGRSETSGE, ESQSAPC, QSTKRSRAQR, NCHCKKDKK, ARKDRGRAGSQNDAE, QKTNPSCIDECGNE, PEESTHH, TERQRAANDYCASE, GPGAGDQTSQ, RADAEGQCADSCKGTETCGGGRSR, and KSASTEC
Turns	DSYCHNRY, VDPNDTFH, AGNNYCQ, and LFNCHCK
Flexibility	IQLRQHR, DIVGGRI, QLCDGGNK, AEVLKPSGRK, NFIKDGV, DTVARKDTGTAS, RCYKKDS, EIECPGGRSETS, ESLLQSTKRSRA, NCHCKKDK, TVVARKDRGRAGS, LDFQKTN, PGAGDQT, QCADSCKGTETCGGGRSRL, and EAMKSAS
Antigenic propensity	IQLRQHRNGL, DSYCHNRYKQLCDGGNKKFCDKT, QSQQTAEWR, LKPSGRKVQ, DPNDTFH, KDGVEKH, DTVARKDTGTASQKAKAWRCYKKDSLTYEARS, PGGRSETSGE, LSQHYTREKELQKVIESQ, LLQSTKRSRAQRF, CHCKKDKKFCTTVVARKDRGRAGSQNDAEWR, EELDFQKTNPSC, VPEESTHH, WTKLPSKIDEATQKFCTERQRAANDY, AGDQTSQFR, KKAAMRADAEGQ, RSRLDGP, and MKSASTE
Exposed surface	QLRQHRN, CHNRYKQL, NKKFCDKT, LKPSGRKV, QKAKAWRCYKKDSLTY, QHYTREKELQKV, QSTKRSRAQR, CHCKKDKKFCT, and ARKDRGRA
Polarity	IQLRQHRNGL, DSYCHNRYKQL, NKKFCDKT, LKPSGRKV, IKDGVEKHCSPF, DTVARKD, QKAKAWRCYKKDS, RSETSGE, SQHYTREKELQKVIE, QSTKRSRAQRF, FNCHCKKDKKFCT, VVARKDRGRAG, RCYSLEELDFQK, VPEESTHHITWTK, QKFCTERQRAAN, QFRCIKKAAMRA, and AHEGISEA

**Table 5:** Forecasted B-cell epitopes through the utilization of the ABCpred server.

**Rank**	**Sequence**	**Start position**	**Score**	**VaxiJen score**	**Allergenicity**	**Water solubility**
1	TASQKAKAWRCYKKDS	180	0.93	0.7433	Nonallergen	Good
2	QQTAEWRCYFAEVLKP	92	0.92	0.6693	Allergen	Good
2	GGEIECPGGRSETSGE	209	0.92	1.5598	Allergen	Good
3	GTGIQSQQTAEWRCYF	86	0.90	0.8636	Allergen	Poor
3	PSCIDECGNELPCQGA	334	0.90	0.6908	Nonallergen	Good
3	VIESQSAPCHDACVAT	239	0.90	0.7236	Nonallergen	Poor
4	ARSICVDNCGGEIECP	200	0.89	0.7898	Nonallergen	Good
5	RCIKKAAMRADAEGQC	409	0.88	1.5158	Nonallergen	Good
5	AGSQNDAEWRCYSLEE	311	0.88	1.0924	Nonallergen	Good
5	LGVVDPNDTFHATAHN	125	0.88	0.3426	Nonallergen	Poor
6	KGTETCGGGRSRLDGP	429	0.87	0.9042	Nonallergen	Good
6	THHITWTKLPSKIDEA	355	0.87	−0.0672	Allergen	Good
7	CQGVLADTVARKDTGT	165	0.86	0.3930	Nonallergen	Good
8	QAELDSYCHNRYKQLC	154	0.85	0.4352	Nonallergen	Good
8	DEATQKFCTERQRAAN	259	0.85	−0.0670	Nonallergen	Good
9	RSRLDGPLSVDDVLNA	438	0.83	0.5145	Nonallergen	Good
9	TERQRAANDYCASEFA	376	0.83	0.1747	Nonallergen	Good
9	DFQKTNPSCIDECGNE	328	0.83	0.2226	Nonallergen	Good
9	QRFIDDACQKLFNCHC	277	0.83	0.0537	Nonallergen	Good
10	NRYKQLCDGGNKKFCD	63	0.82	−0.8381	Nonallergen	Good
10	FADSLARYGPGAGDQT	380	0.82	0.1867	Nonallergen	Good
10	IQLRQHRNGLGVEQSF	23	0.82	0.7074	Nonallergen	Good
10	YFAEVLKPSGRKVQCV	100	0.82	0.8510	Allergen	Good

**Table 6:** Anticipated discontinuous B-cell epitope residues within the MIC13 sequence.

**No.**	**Residues**	**Number of residues**	**Score**
1	A:M1, A:V2, A:G3, A:V4, A:F5, A:Q6, A:L7, A:C8, A:L9, A:L10, A:T11, A:C12, A:L13, A:L14, A:C15, A:L16, A:L17, A:K18, A:G19, A:S20, A:D21, A:A22, A:I23, A:Q24, A:L25, and A:R26	26	0.966
2	A:R29, A:N30, A:G31, A:L32, A:G33, A:V34, and A:E35	7	0.872
3	A:R64, A:Y65, A:Q67, A:L68, A:C69, A:D70, A:G71, A:G72, A:N73, A:K74, A:K75, A:F76, A:C77, A:D78, A:K79, and A:T80	16	0.72
4	A:S274, A:R275, A:F279, A:I280, A:D281, A:D282, A:A283, A:C284, A:Q285, A:K286, A:L287, A:F288, A:N289, A:C290, A:H291, A:C292, A:K293, A:K294, A:D295, A:K296, A:K297, A:F298, A:C299, A:T300, A:T301, A:V302, A:G309, A:R310, A:A311, A:G312, A:S313, A:Q314, A:N315, A:C321, A:S323, A:L324, A:E325, A:E326, A:L327, A:E339, A:C340, A:E353, A:S354, A:T355, A:H356, A:H357, A:I358, A:T359, A:W360, A:T361, A:K362, A:L363, A:P364, A:S365, A:K366, A:D368, A:E369, A:A370, A:T371, A:Q372, A:K373, A:F374, A:C375, A:T376, A:E377, A:R378, A:R380, A:A381, A:A425, A:D426, A:S427, A:C428, A:K429, A:G430, A:T431, A:E432, A:A453, A:H454, A:E455, A:G456, A:S458, A:E459, A:A460, A:M461, A:K462, A:S463, A:A464, A:S465, A:T466, A:E467, and A:C468	91	0.695
5	A:Y85, A:G86, A:T87, A:G88, A:I89, A:Q90, A:S91, A:Q92, A:Q93, A:T94, A:A95, A:E96, A:R98, A:V104, A:L105, A:K106, A:P107, A:S108, A:G109, A:K111, A:V127, A:V128, A:D129, A:P130, A:N131, A:D132, A:T133, A:F134, A:H135, A:A136, A:T137, A:A138, A:H139, A:N140, A:Q141, A:L143, and A:N144	37	0.689
6	A:A160, A:G161, A:N163, A:Y164, A:C165, A:Q166, A:G167, A:V168, A:L169, A:A170, A:D171, A:T172, A:T178, A:G179, A:T180, A:A181, A:S182, A:Q183, A:K184, A:A185, A:K186, A:A187, A:R189, A:C190, A:Y191, A:K192, A:D194, A:S195, A:L196, A:G217, A:R218, A:T221, A:S222, A:G223, A:E224, A:L225, A:L226, A:S227, A:Q228, A:H229, A:Y230, A:T231, A:R232, A:E233, A:K234, A:E235, A:L236, A:Q237, A:K238, A:V239, A:E241, A:S242, A:Q243, A:S244, A:A245, A:P246, A:C247, A:H248, and A:D249	59	0.677
7	A:D263, A:T264, and A:E265	3	0.593

**Table 7:** Percentile rank related to MIC13 binding to MHC-I molecules.

**Allele**	**Start–end**	**Peptide sequence**	**Percentile rank**	**Immunogenicity**
H-2-Db	252–261	VATPINPPIC	0.73	0.1827
	349–358	AVPEESTHHI	1.3	0.11629
	452–461	NAHEGISEAM	1.5	0.22548

H-2-Kb	227–236	SQHYTREKEL	2.6	0.06573
	354–363	STHHITWTKL	3.3	0.32572
	23–32	IQLRQHRNGL	3.5	0.03024

H-2-Kk	231–240	TREKELQKVI	0.27	−0.36472
	41–50	EETDIVGGRI	0.39	0.29546
	349–358	AVPEESTHHI	1.8	0.11629

H-2-Dd	349–358	AVPEESTHHI	0.92	0.11629
	442–451	DGPLSVDDVL	1.6	−0.07194
	310–319	RAGSQNDAEW	1.8	−0.16217

H-2-Kd	59–68	SYCHNRYKQL	0.5	−0.19401
	133–142	TFHATAHNQI	0.6	0.08308
	278–287	RFIDDACQKL	0.68	−0.14929

H-2-Ld	155–164	SPFQMAGNNY	0.5	−0.1888
	349–358	AVPEESTHHI	1.7	0.11629
	354–363	STHHITWTKL	2.3	0.32572

**Table 8:** Percentile rank related to MIC13 binding to MHC-II molecules, IFN-*γ* induction, IL-4 induction, and antigenicity.

**Allele**	**HTL epitope**	**Percentile rank**	**Antigenicity**	**IFN-*γ* inducing**		**IL-4 inducing**	
				**Result**	**Score**	**Result**	**SVM score**
H2-IAD	REKELQKVIESQSAP	0.69	0.6014	Positive	0. 0493	Positive	0.31
H2-IAD	VARKDTGTASQKAKA	1.40	0.7749	Positive	0.1854	Negative	−0.08
H2-IAD	NAHEGISEAMKSAST	1.60	0.2539	Positive	0.3223	Positive	0.30
H2-IAB	ASEFADSLARYGPGA	1.50	0.4525	Positive	0.2660	Positive	1.69
H2-IAB	VATPINPPICADTES	1.70	11552	Positive	0.3551	Negative	0.11
H2-IAB	ETDIVGGRISSLQAE	5.20	11691	Negative	1	Positive	0.47
H2-IED	TSQFRCIKKAAMRAD	0.39	0.8544	Negative	−0.2046	Positive	0.31
H2-IED	SQHYTREKELQKVIE	0.81	0.6225	Positive	0.2132	Positive	0.32
H2-IED	DKKFCTTVVARKDRG	0.88	0.8768	Negative	2	Positive	0.47

**Table 9:** Forecasted MIC13 epitopes using CTLpred.

**Peptide rank**	**Start position**	**Sequence**	**Score (ANN/SVM)**	**Immunogenicity**
1	49	RISSLQAEL	0.98/1.0524939	−0.24849
2	200	ARSICVDNC	0.77/0.99567172	0.08152
3	138	AHNQILNFI	0.97/0.76815731	0.06344

## Data Availability

The data used to support the findings of this study is available from the corresponding author upon reasonable request.

## Supporting Information

Supporting Information Additional supporting information can be found online in the Supporting Information section. The links of all bioinformatics online servers used in the research are embedded in Table S1.
