# Supplementary material for: MIC13 of Toxoplasma gondii: Potential Gene for Vaccine Candidate—An In Silico Approach
Source: J Parasitol Res. 2025 Sep 26;2025:3114690. doi: 10.1155/japr/3114690 (PMC12494477; doi:10.1155/japr/3114690)
Supplement: Supporting Information — Additional supporting information can be found online in the Supporting Information section. The links of all bioinformatics online servers used in the research are embedded in Table S1. [file 3114690.f1.docx]

**Supplementary Table 1.** The links of all bioinformatics online servers used in the research

| Online site | Link |
| --- | --- |
| NCBI | <https://www.ncbi.nlm.nih.gov/protein/> |
| Expasy ProtParam | <https://web.expasy.org/protparam/> |
| ANTIGENpro | <http://scratch.proteomics.ics.uci.edu/> |
| VaxiJen v. 2.0 | <http://www.ddg-pharmfac.net/vaxijen/VaxiJen/VaxiJen.html> |
| AllergenFP v.1.0 | <https://ddg-pharmfac.net/AllergenFP/> |
| AlgPred | <https://webs.iiitd.edu.in/raghava/algpred/submission.html> |
| SOLpro | <http://scratch.proteomics.ics.uci.edu/> |
| SignalP | <http://www.cbs.dtu.dk/services/SignalP/> |
| NetPhos 3.1 | <http://www.cbs.dtu.dk/services/NetPhos/> |
| GPS-PAIL 2.0 | <http://pail.biocuckoo.org/> |
| NetOGlyc 4.0 | <http://www.cbs.dtu.dk/services/NetOGlyc/> |
| NetNGlyc 1.0 | <http://www.cbs.dtu.dk/services/NetNGlyc/> |
| TMHMM ver. 2.0 | <http://www.cbs.dtu.dk/services/TMHMM-2.0/> |
| PSORT II | <http://psort.hgc.jp/form2.html> |
| GOR IV | <https://npsa-prabi.ibcp.fr/cgi-bin/npsa_automat.pl?page=npsa_gor4.html> |
| DIpro | <http://scratch.proteomics.ics.uci.edu/> |
| GalaxyRefine | <http://galaxy.seoklab.org/cgi-bin/submit.cgi?type=REFINE> |
| SWISS-MODEL | <https://swissmodel.expasy.org/> |
| ProSAweb | <https://prosa.services.came.sbg.ac.at/prosa.php> |
| ABCpred | <http://crdd.osdd.net/raghava/abcpred/> |
| Bcepred | <http://crdd.osdd.net/raghava/bcepred/bcepred_submission.html> |
| BCPREDS 1.0 | <http://ailab-projects2.ist.psu.edu/bcpred/predict.html> |
| ElliPro | <http://tools.iedb.org/ellipro> |
| IEDB | <http://tools.iedb.org/bcell/> |
| PepCalc | <https://pepcalc.com/> |
| MHCI | <http://tools.iedb.org/mhci/> |
| MHCII | <http://tools.immuneepitope.org/mhcii> |
| IFNepitope | <https://webs.iiitd.edu.in/raghava/ifnepitope/predict.php> |
| IL4-pred | <https://webs.iiitd.edu.in/raghava/il4pred/design.php> |
| CTLpred | <http://www.imtech.res.in/raghava/ctlpred/index.html> |
| C-ImmSim | <https://kraken.iac.rm.cnr.it/C-IMMSIM/index.php> |
